# Profiles of Steroid Hormones in Canine X-Linked Muscular Dystrophy via Stable Isotope Dilution LC-MS/MS

**DOI:** 10.1371/journal.pone.0126585

**Published:** 2015-05-26

**Authors:** Helio A. Martins-Júnior, Rosineide C. Simas, Marina P. Brolio, Christina R. Ferreira, Felipe Perecin, Guilherme de P. Nogueira, Maria A. Miglino, Daniele S. Martins, Marcos N. Eberlin, Carlos E. Ambrósio

**Affiliations:** 1 ThoMSon Mass Spectrometry Laboratory—Institute of Chemistry, University of Campinas (UNICAMP), Campinas, São Paulo, Brazil; 2 AB SCIEX of Brazil, São Paulo, São Paulo, Brazil; 3 DAPSA, Faculty of Veterinary Medicine, São Paulo State University-UNESP, Araçatuba, São Paulo, Brazil; 4 Faculty of Animal Sciences and Food Engineering—FZEA, University of São Paulo—USP, Pirassununga, São Paulo, Brazil; 5 Faculty of Veterinary Medicine and Animal Science—FMVZ, University of São Paulo—USP, São Paulo, São Paulo, Brazil; 6 Department of Chemistry, Purdue University, West Lafayette, Indiana, United States of America; Western University of Health Sciences, UNITED STATES

## Abstract

Golden retriever muscular dystrophy (GRMD) provides the best animal model for characterizing the disease progress of the human disorder, Duchenne muscular dystrophy (DMD). The purpose of this study was to determine steroid hormone concentration profiles in healthy golden retriever dogs (control group - CtGR) versus GRMD-gene carrier (CaGR) and affected female dogs (AfCR). Therefore, a sensitive and specific analytical method was developed and validated to determine the estradiol, progesterone, cortisol, and testosterone levels in the canine serum by isotope dilution liquid chromatography coupled with tandem mass spectrometry (LC-MS/MS). To more accurately understand the dynamic nature of the serum steroid profile, the fluctuating levels of these four steroid hormones over the estrous cycle were compared across the three experimental groups using a multivariate statistical analysis. The concentration profiles of estradiol, cortisol, progesterone, and testosterone revealed a characteristic pattern for each studied group at each specific estrous phase. Additionally, several important changes in the serum concentrations of cortisol and estradiol in the CaGR and AfCR groups seem to be correlated with the status and progression of the muscular dystrophy. A comprehensive and quantitative monitoring of steroid profiles throughout the estrous cycle of normal and GRMD dogs were achieved. Significant differences in these profiles were observed between GRMD and healthy animals, most notably for estradiol. These findings contribute to a better understanding of both dog reproduction and the muscular dystrophy pathology. Our data open new venues for hormonal behavior studies in dystrophinopathies and that may affect the quality of life of DMD patients.

## Introduction

Golden retriever dogs are affected by a degenerative muscle disease that is genetically homologous to Duchenne muscular dystrophy (DMD) in humans and are thus commonly used as an animal model for this disease. As in humans, golden retriever muscular dystrophy (GRMD) affects the expression of the dystrophin gene, which codes for a cytoskeletal protein and results in a weakening of the musculoskeletal system and impaired locomotion. [1–2].

The absence of dystrophin protein in GRMD results from a frame-shifting point mutation in the canine dystrophin gene, whereas deletions are the most frequent mutations in DMD patients [3–4]. Similar to DMD patients, GRMD dogs suffer from repeated cycles of muscle necrosis and regeneration followed by fibrosis, postural abnormalities, respiratory or heart failure, and premature death [5,6,7]. The close resemblance of GRMD dogs to DMD patients, with respect to both body weight and pathological expression of the disease [7], has made them an ideal model to study the pathogenic mechanisms of DMD [8,9,10] and the effect of therapeutic interventions, such as stem cells or specific drugs [11–12]. However, changes in steroid hormone levels in GRMD dogs compared with healthy dogs are unknown. This information may be crucial for further research into the effect of this dystrophin deletion on the physiology of these animals.

The reliable measurement of steroid hormones in humans is a powerful diagnostic tool for assessing their hormonal status and investigating endocrine disorders. For the last thirty years, the predominant method for measuring the levels of circulating hormones has been the radioimmunoassay (RIA) [13]. However, the RIA method for steroid hormone quantification presents limitations, such as a lack of commercially available kits that are specific for dogs. Additionally, because steroid hormones are small molecules with similar molecular structures and are present in sub micromolar or nanomolar concentrations, high levels of uncertainty and cross-reaction are expected to occur with the use of RIA methods [14]. Liquid chromatography tandem mass spectrometry (LC-MS/MS) is currently considered the gold standard analytical platform to determine the steroid hormone levels in blood and other biological fluids [15]. When compared with RIA, LC-MS/MS offers the following key advantages: identification based on molecular structure, simultaneous quantification of multiple analytes, high sensitivity, broad linear dynamic range, and a reduced matrix effect [16]. LC-MS/MS with stable isotope dilution also benefits from the improved selectivity and sensitivity afforded by the inclusion of stable (heavy) isotope-labeled internal standards, which, except for their mass, are nearly identical analogs of the targets. Isotopes present nearly identical LC retention times and MS/MS chemistries. The procedure for employing these stable isotope-labeled internal standards involves spiking them into a sample at a known concentration. The response ratio between the analytes and the corresponding labeled compounds is then interpolated onto a standard curve to calculate the absolute amount of analyte in the unknown sample. The level of specificity achieved using this method is superior to any other bioanalytical technique employed for biomarker analysis [17].

Steroids hormones administrations are commonly therapy to control the dystrophy evolution. The standard-of-care drug for young dystrophic boys is corticosteroid prednisone. However a recent article in murine model of dystrophy highlighted the importance of tested different steroids in body [18]. This blood test showed with mass spectrometry could help other researchers to develop pharmacological therapies and certifications of efficacy.

The uterus of female model of canine dystrophy was studied showing a weakness of structure ad had collagen alteration and muscle fiber commitment in affect tissue [19].

In this study, we aimed to determine the steroid hormone profiles of healthy golden retriever dogs (control group—CtGR) versus GRMD-gene carrier (CaGR) and affected female dogs (AfCR) and to correlate these profiles to the disease condition. To achieve this goal, we carefully developed and validated a specific LC-MS/MS method for the simultaneous determination of four target steroid hormones (i.e., progesterone, estradiol, cortisol, and testosterone) in canine serum. We evaluated this method by systematically testing the critical parameters of equilibration time, sensitivity, linearity, precision, recovery, carryover, matrix effects, and selectivity (matrix interference) and applied it to assess and compare the steroid hormone status of GRMD and control groups during the estrous phase of the reproductive cycle.

## Materials and Methods

### Animals

Twenty-three female golden retriever dogs among two to five years of age provided blood samples for use in this study. All study procedures were performed in accordance with the ethical principles of animal research approved by the ethics committee for the use of animals at the Faculty of Veterinary Medicine and Animal Science of the University of São Paulo (protocol number 2282/2011). The animals were classified into three groups according to their genetic condition, as follows: group #1: CtGR—control golden retriever, consisting of thirteen healthy females with an average body weight of 28.7 ± 4.5 kg; group #2: CaGR—GRMD-gene carrier animals (no muscular dystrophy clinical symptoms but dystrophin gene mutation present), consisting of six animals with an average body weight of 26.9 ± 2.1 kg; and group #3: AfGR—affected golden retriever, consisting of four animals carrying the dystrophin gene mutation that were clinically affected, with an average body weight of 20.7 ± 4.5 kg. The animals from groups CaGR and AfGR belong to the GRMD colony established in the Department of Surgery, Faculty of Veterinary Medicine and Animal Science of the University of Sao Paulo (FMVZ-USP). The healthy animals constituting the CtGR group belong to kennel Chasse, located in Cotia, Sao Paulo, Brazil. A total of 153 samples from these three experimental groups were used. Samples from all experimental groups were further subdivided into four groups according to the specific stage of the estrous cycle (i.e., anestrus, proestrus, estrus, and diestrous) the animal was in when the samples were collected.

Serial vaginal cytology examinations were used to determine the phase of the estrous cycle of each animal at the time of blood collection. All females were observed for at least 10 calendar days prior to determination of estrous phase. The vaginal samples were collected and analyzed according to routine protocols used in reproduction in dogs.

### Serum Specimens

Blood sample collection was performed by left or right animal cephalic vein puncture. An approximate volume of 4 mL of blood was collected in silicon-coated glass tubes containing clot accelerator (SiO_2_). The samples were centrifuged at 1500 rpm for 8 minutes, and the serum was stored at—20°C until further processing and laboratory analysis.

### Chemicals and the preparation of calibrators

HPLC-grade methanol, isopropanol, and methyl tert-butyl ether (MTBE) were purchased from J. T. Baker (Phillipsburg, NJ, USA). Acetone was acquired from Labsynth (Diadema,SP, Brazil), and all ultrapurified water was generated on-site using a Milli-Q 3 Ultrapure Water System (Merck-Millipore, Darmstadt, Germany). The steroid standards were acquired from Sigma-Aldrich (St. Louis, MO, USA). The internal standards cortisol-*d*_4_, testosterone-^13^ *C*_*3*_, and estradiol-*d*_*2*_ were purchased from Sigma Aldrich (St. Louis, MO, USA) and progesterone-*d*_9_ was purchased from Steraloids Inc (Newport, RI, USA).

Standards’ stock solutions for all of the steroids and their respective internal standards were gravimetrically prepared for calibration. A mass of 1.0 to 3.0 mg of each hormone was accurately weighed and dissolved in 15.0 mL of methanol to prepare solutions ranging from 24.63 to 70.42 μg g^-1^. The working and calibration solutions were prepared accordingly by dilutions with methanol. Six different concentration levels were used for the calibration curves, and three recovery levels were prepared daily and analyzed during three sequential days to evaluate the method recovery and performance.

### LC-MS/MS Instrumentation

The LC-MS/MS quantitation was performed on a hybrid triple quadruple/linear ion trap mass spectrometer QTRAP 5500 (AB SCIEX, Concord, Canada) equipped with a 10 eV krypton lamp PhotoSpray source (atmospheric pressure photoionization—APPI). The mass spectrometer was coupled with an Agilent 1200 HPLC system (Agilent Technologies, Waldbronn, Germany) consisting of a high performance autosampler, thermostatted column compartment, and binary and isocratic pumps for mobile phase gradient and APPI dopant deliveries, respectively. The quantitation was performed via multiple reaction monitoring (MRM) and the optimized source parameters included nebulizing gas at 55 psi (GS1), auxiliary (lamp) gas at 20 psi (GS2), curtain gas at 10 psi (CUR), vaporization temperature of 400°C (TEM), and ion transfer voltage (IS) at—750 V and 830 V for the negative and positive ion modes, respectively. The collision-induced dissociation (CID) experiments were performed with optimized collision gas pressure at 10 a.u. (set point medium). The dopant-assisted APPI analysis was performed with an optimized flow of toluene at 120 μL min^-1^.

The APPI(+)-MS/MS experiments were composed of 12 MRM transitions with a 25 ms dwell time and a 5 ms pause time. All selected transitions corresponded to fragmentation of the protonated molecules [M + H]^+^ of cortisol, cortisol-*d*_*4*_, testosterone, testosterone-^13^ *C*_*3*_, progesterone, and progesterone-*d*_*9*_. For the APPI(-)-MS/MS experiments, 4 MRM transitions were monitored at 100 ms dwell time with a 5 ms pause time for the deprotonated molecules [M—H]^–^ of estradiol and its internal standard (estradiol-*d*_*2*_ *)*. A settling time of 50 ms was used for simultaneous polarity switching, resulting in a total method cycle of 880 ms. The MRM transitions were optimized by the infusion of standard solutions of all compounds at a flow rate of 10 μL min^-1^ and an HPLC sheath flow of 100 μL min^-1^ of methanol. **Table 1**presents the optimized parameters for the monitored quantifier and qualifier MRM transitions.

**Table 1:** Mass spectrometer MRM parameters optimized for the method.

Hormone	Q1	Q3	Purpose	Voltages (V)			Polarity	Ion Ratio
	(*m/z*)	(*m/z*)		DP	CE	CXP		
Estradiol	271.0	143.1	Quantifier	-110	-54	-13	–	1.19
		145.1	Qualifier		-55	-12		
Estradiol-*d*_*2*_	273.0	185.1	IS_Quantifier	-110	-56	-15	–	0.59
		145.1	IS_Qualifier		-54	-13		
Cortisol	363.2	121.2	Quantifier	45	41	16	+	1.12
		327.3	Qualifier		23	22		
Cortisol-*d*_*4*_	367.2	121.2	IS_Quantifier	45	41	16	+	1.46
		331.3	IS_Qualifier		20	22		
Progesterone	315.0	97.3	Quantifier	60	35	12	+	2.42
		109.1	Qualifier		27	16		
Progesterone-*d*_*9*_	324.2	100.1	IS_Quantifier	60	33	12	+	1.38
		113.1	IS_Qualifier		29	16		
Testosterone	289.1	97.0	Quantifier	45	29	10	+	1.88
		109.1	Qualifier		27	4		
Testosterone-^*13*^ *C*_*3*_	292.0	100.0	IS_Quantifier	45	29	18	+	0.69
		112.1	IS_Qualifier		27	10		

DP = Declustering Potential; CE = Collision Energy; CXP = Collision Cell Exit Potential; IS = Internal Standard

The software packages Analyst 1.5.1, PeakView 1.2, MultiQuant 2.1, and MarkerView 1.2.1 (AB SCIEX, Concord, Canada) were used, respectively, for mass spectral data acquisition, processing, quantitation and principal component analysis (PCA). The data quantitation was performed using the chromatographic peak areas after integration with the MultiQuant MQ4 algorithm. The calibration curves were built by linear regression analysis using the least-squares method with 1/x or 1/x^2^ weighting to prioritize the calibration points with lower hormone concentrations.

### Sample preparation and chromatographic conditions

The hormones were extracted from 450 μL of serum after spiking with 15 μL of internal standards’ solution and vortexing for 10 seconds. A two-step liquid/liquid extraction with 500 μL each of methyl tert-butyl ether (MTBE) was performed for 1 min using a vortex and transferring the top organic layer into a new tube. The sample extracts were centrifuged at 10,000 rpm for 10 min and then evaporated to dryness at room temperature with nitrogen flow. The residues were reconstituted with 150 μL of methanol, vortexed for 20 s, and transferred into an autosampler vial containing a 250 μL insert. The HPLC injection volume was set to 50 μL. The hormone separation was achieved using a Kinetex Pentafluorophenyl (PFP) column (2.6 μm, 100 x 4.6 mm), and the mobile phase consisted of ultrapurified water (phase A) and methanol (phase B) with no additives. The mobile phase flow rate was 400 μL min^-1^ and the column oven and autosampler temperatures were set to 45°C and 15°C, respectively. The separation gradient was defined as follows: the initial composition (82% phase B) was held during 1.5 min and then ramped to 100% phase B in 1.0 min; the composition of 100% organic was held until 5.0 min, changed back to 82% in 0.1 min, and remained at this composition until the end of chromatographic run. The total acquisition time was 6.5 min, with a post-run equilibration time of 2.0 min. Acetone was used as the needle and needle-seat flushing solvent for 15 s after sample aspiration.

### Statistical Analysis

The MarkerView 1.2.1 software (AB SCIEX, Concord, Canada) was used for multivariate principal component analysis with discriminant analysis (PCA-DA) of the LC-MS/MS data. The MedCalc software (MedCalc Software bvba, Mariakerke, Belgium) was used for parametric statistics, and the data presented here are shown as the mean and the standard deviations. The Tukey test was used to detect outliers, and the D’Agostino-Pearson test was used to verify that the data were normally distributed with no logarithm transformation. The differences among sample groups under study were analyzed using Student’s t-test, and P < 0.05 was considered a statistically significant difference. The interaction tests were performed using the Action 2.4 software.

## Results

### Method performance and validation

**Fig 1:**
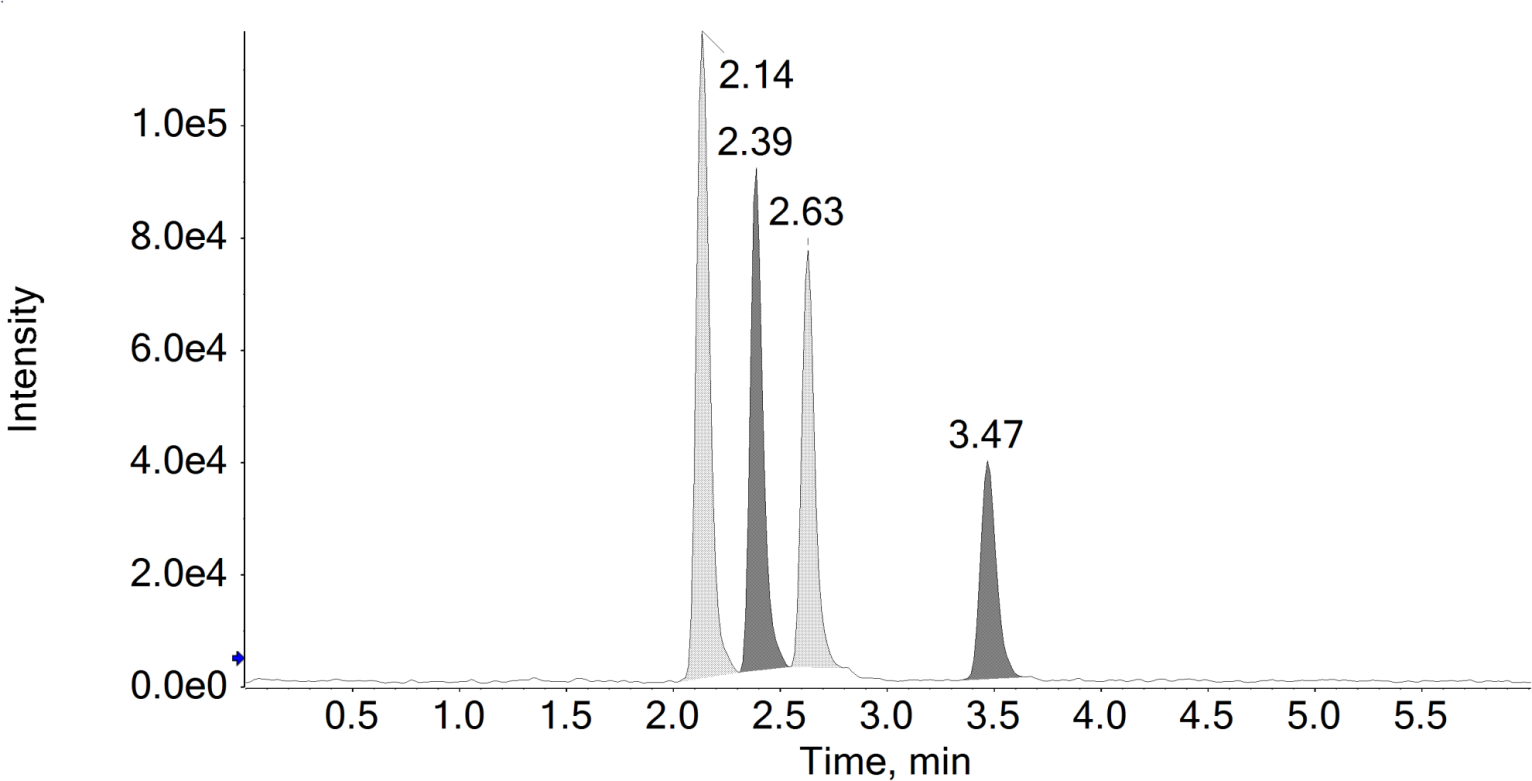
Representative Total Ion Chromatogram (TIC) obtained for the hormones. The elution sequence from left to right is cortisol (2.14 min), estradiol (2.39 min), testosterone (2.63 min), and progesterone (3.47 min).

Isotopic dilution was used to accurately quantify all hormones determined by LC-MS/MS in the canine serum samples. A pool of serum samples was obtained and properly homogenized for use as the matrix in the method development and validation. The method was evaluated by testing critical parameters such as equilibration time, sensitivity, linearity, precision, recovery, carryover, matrix effects, and selectivity (matrix interference). Fig 1 shows a representative total ion chromatogram (TIC) obtained for the hormones in simultaneous positive and negative APPI mode. Two MRM transitions were monitored for each analyte, as were the internal standards. The second MRM transition was used as for confirmatory purposes by measuring the ion ratio obtained between areas of two transitions. The abundance ratios for the ions of the internal standards transitions were also calculated and verified to confirm that no coeluted matrix interferences had affected the quantitative data accuracy. The maximum accepted ion abundance ratio variation between areas for all analytes and internal standards transitions was 20% [20].

### Sample Preparation

The hormone concentrations were determined first for the serum pool used as the matrix. After a single analysis of the serum hormone content, a set of five samples was extracted with different added concentrations of the internal standards, which ranged from approximately 80% to 120% of the expected hormone concentration. The acquired data were evaluated to determine the endogenous concentration of all hormones that corresponded to a 1:1 ratio with the respective internal standard. Once the endogenous concentrations were determined, three sets of recovery tests were carried out at concentration levels corresponding to 1.0, 1.5, and 2.0 times the endogenous concentration.

### Equilibration time, recovery, and precision

Four aliquots of serum were analyzed to investigate the equilibration time between hormones and labeled internal standards at 0.0, 0.5, 1.0, and 1.5 h after extraction. No significant differences were observed from 0.5 to 1.5 h in the 1:1 ratio between analytes and internal standards, suggesting that the equilibration time is achieved by 0.5 h. The percent recovery was calculated by comparison of area ratios (analyte/internal standard) provided for extracted samples versus post-extraction spiked samples at the same nominal concentration. The intra- and inter-assay maximum imprecision was smaller than 7%, ranging from recoveries of 96.5% (cortisol) to 106.5% (estradiol). Table 2 shows the hormone recoveries and imprecision.

**Table 2:** Summary of method performance and main results.

Hormone	Level (pg mL^-1^)	Intra-assay (*n* = 3)				Inter-assay (*n* = 9)				LOD (pg mL^-1^)	LOQ (pg mL^-1^)
		Mean (pg mL^-1^)	SD (pg mL^-1^)	RSD (%)	Recovery (%)	Mean (pg mL^-1^)	SD (pg mL^-1^)	RSD (%)	Recovery (%)		
Estradiol	273.4	268.8	1.88	0.7	98.3	269.8	4.05	1.5	98.7	2.0	5.0
	410.1	436.8	10.0	2.3	106.5	426.9	15.8	3.7	104.1		
	546.8	570.3	7.41	1.3	104.3	561	16.3	2.9	102.6		
Cortisol	630	620	11.2	1.8	98.3	614	19.0	3.1	96.5	3.0	10.0
	950	964	54.0	5.6	100.9	944	45.3	4.8	98.6		
	1260	1282	52.6	4.1	101.7	1342	83.2	6.2	106.2		
Progesterone	890	910	21.8	2.4	102.7	913	28.3	3.1	102.4	3.0	8.0
	1340	1364	30.0	2.2	101.8	1358	36.7	2.7	101.1		
	1780	1732	36.4	2.1	97	1766	33.6	1.9	98.8		
Testosterone	48.6	50.9	2.60	5.1	104.8	50	1.95	3.9	102.9	10.0	25.0
	72.9	70.4	2.96	4.2	96.6	71.9	3.31	4.6	98.7		
	97.2	99.9	3.70	3.7	102.8	100.6	2.82	2.8	103.5		

### Sensitivity and Linearity

The limit of quantitation (LOQ) for each compound was calculated as the lowest hormone concentration that could be determined with a signal-to-noise ratio of 10. The limit of detection (LOD) was determined as the minimum injected amount of the hormone that produced a signal-to-noise ratio of 3. No smoothing algorithm was applied to the chromatograms before LOD and LOQ estimation. The noise values were considered in the calculations to be three times the baseline standard deviation obtained at approximately 30 seconds before the respective analyte retention time.

To evaluate the linearity of the calibration curve, six to eight calibration points were obtained after injections in a concentration range from the LOQ to one thousand times the LOQ. All hormones response ratios showed a linear dynamic range greater than 3 orders of magnitude and r^2^ values equal to or greater than 0.99.

### Matrix effect and carryover

APPI is an ionization technique that is much less subject to ion suppression than other API techniques, such as electrospray or APCI. With the use of isotopic dilution, it is commonly supposed that the analyte and its internal standard will show identical ionization effects and be subjected to the same ion suppression or ion enhancement effects. These assumptions are only valid in the linear measurement range where ionizable molecules do not cause ion source saturation [21]; thus, matrix effects were not investigated. However, the carryover effect was investigated, and an appropriate autosampler cleaning program was necessary and configured until no amount of any of the steroids, especially progesterone, was detected between injections. A 15-s acetone rinsing program was set to avoid progesterone carryover between injections, and the mobile phase was allowed to pass through the injection valve for the duration of the run, including the highest organic composition. Following the elution of the last analyte, the injection valve was configured for multiple switches between the main and bypass positions to improve injection system clean up.

### Principal Component Analysis (PCA)

**Fig 2:**
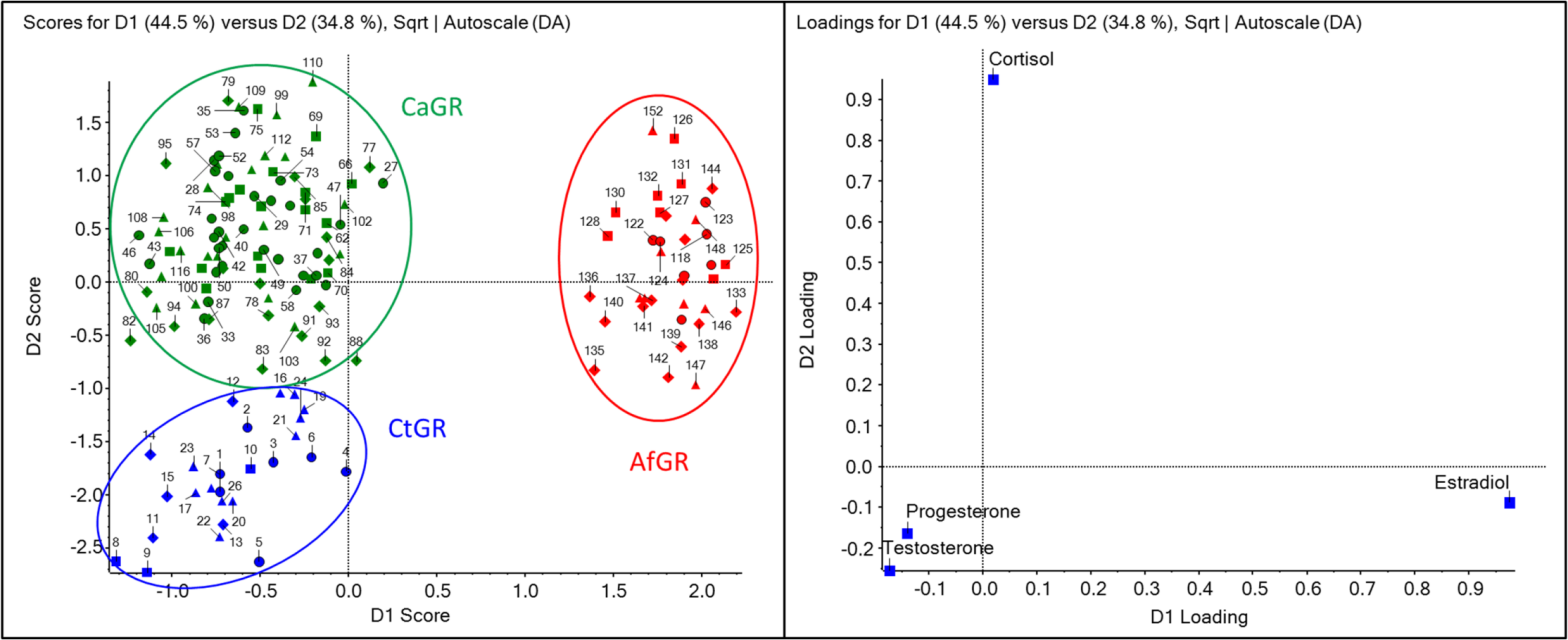
PCA-DA score (left) and loading (right) plots for all serum samples studied. Data collection of CtGR: control golden retriever (data in blue); CaGR: carrier golden retriever (data in green), and AfGR: affected golden retriever (data in red). Each symbol represents an estrous phase: anestrus (circle), proestrus (square), estrus (rhombus), and diestrus (triangle).

The quantitative LC-MS/MS data were first analyzed by multivariate analysis. The principal component analysis (PCA) is a statistical method that reduces the dimensionality of the acquired data by variables (principal components). This methodology reveals group sample relationships and similarities based on their spatial proximity. When used as a supervised method, PCA with discriminant analysis (PCA-DA) allows the classification of samples into known groups. This classification procedure yields the maximum separation between known classes of samples and makes them easier to be distinguished. Fig 2 shows the score and loading plots for all samples, where each point represents a single dog serum sample.

The three sample clusters that are circled and positioned in different areas of the PCA-DA score plot discriminate differences between sample groups as a function of the hormone dosed concentrations. The t-test for between groups differences revealed statistically significant differences between all groups, except for progesterone and estradiol in the CtGR and CaGR groups, where P > 0.05. The results indicate that the major separation between CtGR and AfGR groups in dimension 1 (D1) was accounted for by the estradiol concentration. In the second separation dimension (D2), the dosed levels of progesterone and cortisol were responsible for the separation between CtGR and CaGR.

### Quantitative results

**Fig 3:**
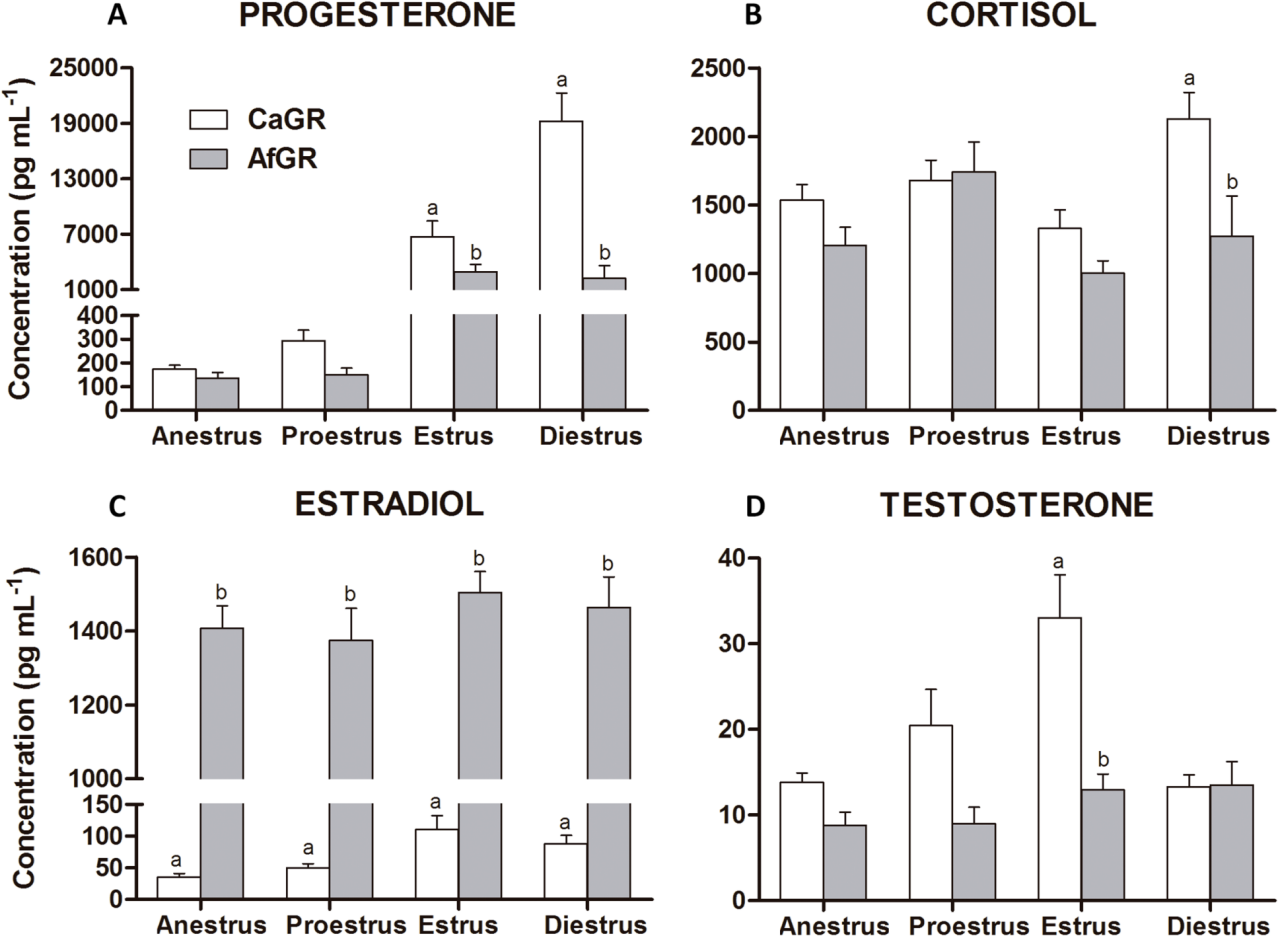
Plasmatic levels (pg mL^-1^) hormones at studied affect group. Progesterone (A), cortisol (B), estradiol (C), and testosterone (D) throughout the reproductive phases (anestrus, proestrus, estrus, and diestrus) of golden retrievers control (CR), carrier of affected gene (CaGR) or GR affected by muscular dystrophy (AfGR). Different letters (a, b, c) above the bars indicate a significant difference between groups (p<0.05). Bars depict the mean, and whiskers depict SEM.

At Fig 3 there is a comparison of the average concentration of hormones in the studied groups. Consistent with the spatial separation observed in the PCA-DA loading plot, the concentration levels of estradiol fully distinguished AfGR from CaGR. Irrespective of the phase of the estrous cycle, the mean concentration of estradiol always exceeded 1,300 pg mL^-1^ in the AfGR group, was different (P < 0.0001) compared with the CaGR group, and demonstrated discrete variation through the estrous period. Fig 3 compares the average hormone concentrations during all estrous periods. The average concentrations of serum cortisol for CaGR and AfGR ranged between 1000 and 2000 pg mL^-1^ in all estrous periods, but the cortisol concentrations were different (P < 0.05) between groups during the diestrus period.

During anestrus and proestrus, the progesterone concentrations were similar between groups; however, during estrus and diestrus, the concentrations of all quantified hormones were significantly different between the CaGR and AfGR groups. Similar trends were observed for testosterone concentrations during estrus, which were significantly different between the CaGR and AfGR groups (P < 0.05).

To better understand both the high levels of estradiol in AfGR across the entire estrous cycle and the higher observed concentrations of cortisol in the CaGR and AfGR groups, interaction and main effects tests were performed to correlate these observations with groups and estrous. The results revealed an interaction between cortisol serum concentrations and animal estrous period, but no interaction was found between the constant high levels of estradiol and the estrous period of the animal.

## Discussion

The principal aim of the current study was to develop the first specific LC-MS/MS assay for the quantification of progesterone, estradiol, cortisol, and testosterone in dogs. The motivation for this goal was the importance of precision and the confident quantification of steroid hormones in dogs for understanding the reproductive changes observed in GRMD animals. This multi-hormone assay was then applied to 153 plasma samples collected from 23 female golden retriever dogs throughout their entire estrous cycle (i.e., anestrous, proestrus, estrus, and diestrus). We observed specific patterns in the concentrations of estradiol, cortisol, progesterone, and testosterone that are characteristic of and distinguish the CtGR, CaGR, and AfGR groups across phases of the estrous cycle. Therefore, the accurate steroid hormone profiling of these experiments seems related to the pathological status associated with GRMD. For the first time we are showing the hormone profile in normal dogs using LC-MS/MS assay using comparison of hormones in carrier and affected females of canine muscular dystrophy.

The observed cortisol concentration differences among the three studied have been observed previously by our group using RIA (unpublished data). However, because differences in accuracy between LC-MS/MS and RIA techniques are widely known, we have chosen to develop an LC-MS/MS method that can provide both lower LOD/LOQ and more reliable data while circumventing the RIA limitation of cross reactivity between steroids and the RIA antibody [22–25].

The higher cortisol levels observed for CtGR should be interpreted with caution because stressful situations are known to induce variations in the concentration of this hormone in various species, including dogs [26–28]. Cortisol concentrations may increase by a factor of 20 above the baseline circulating levels due to stress [29]. We must highlight the altered cortisol level caused by caregiving mother of dysphophic children confirmed the alterations of sexual compromises and sleep [30].

Moreover, the elevated cortisol levels may be explained by a reduction of lean body mass, which is known to increase plasma concentrations of circulating cortisol and is a principal symptom of the disease in affected dogs. In fact, the cortisol concentrations showed an interaction with the animal’s estrous cycle and presented significant differences between the groups during diestrus. We hypothesize that the high cortisol levels might also be associated with the exposure to stressors during prolonged kenneling time in the control animals [31–32]. However, this issue requires further study to clearly evaluate the pathological relevance of such cortisol levels and future experimental designs must incorporate some criteria for the control of stress conditions inside the kennel environment.

Significant differences in the estradiol concentrations were observed between AfGR and the other group. Additionally, the average estradiol concentration was significantly higher in AfGR than it was in all other groups. Interestingly, the estradiol concentrations in the AfGR group also showed very low variability across the estrous cycle, including during anestrus. The observed hyperestrogenism of the AfGR group presented an interaction with the animals in the estrous phase.

During the experiment design, animals with polycystic ovaries were carefully excluded from experimentation because this syndrome is commonly associated with hyperestrogenism [33]. Therefore, the elevated levels of estradiol might be associated with an induction of aromatase because the dosed testosterone levels in AfGR were lower than other groups during most estrous phases [34–35]. Dogs with muscular dystrophy have a liver metabolism overload condition that causes the alanine amino transferase (ALT) concentration to increase in serum, reflecting the damage and loss of liver function [29,36]. We suggest that the elevated estradiol levels observed in this study are related to decreased liver function, which effectively increases the activity of estrogens in the body and has the potential to result in lower fertility. Liver involvement must have careful attention in dystrophy models and patients [37].

At the estrus stage, luteinization of granulosa cells occurs in mature follicles. These follicles start to produce progesterone, and the increased levels of this hormone are detectable in the blood of mammals. This hormonal shift initiates positive feedback at the level of the hypothalamus and pituitary gland, resulting in the secretion of follicle stimulating hormone (FSH) and the pre-ovulatory surge, which is characterized by a sharp peak in the concentration of progesterone during estrus and diestrus [38]. During estrus and diestrus, progesterone was within the expected levels because this hormone is secreted in the last phase of the estrous cycle. However, the significant difference between CaGR and AfGR is due to the metabolism of this hormone performed by the liver. Although the large increase in serum concentrations of ALT is a characteristic of dystrophic dogs, it is not associated with primary causes of damage and loss of liver function; however, it is believed to result from an overload on organ function due to muscular dystrophy [36]. The same imbalance occurs in testosterone concentration because the liver rapidly converts testosterone molecules that are not bound in the tissues.

According to Busch (2004), [39], ALT is essentially a specific liver enzyme for the dog. Increases in ALT serum activity are a general indication of damage to hepatocytes, which release this enzyme into the circulation. Therefore, the measurement of this enzyme is considered the best test for the determination of liver injury; however, we assume that it is not a test of liver function.

Similar to ALT, the serum levels of creatinine kinase (CK) and aspartate aminotransferase (AST) are markedly elevated in GRMD dogs. The level of these enzymes peaked in dogs at four months of age, indicating muscle necrosis, a feature of dystrophic myopathy. However, the urea and creatinine levels are within the laboratory reference ranges. Previous studies indicate that hematologic and serum biochemical analyses, including ALT dosage in female carriers of the GMRD gene, are within the normal range for the species and do not differ from healthy animals [12].

The liver performs the major task of steroid inactivation by removing free steroids from the circulation. Metabolism of steroids typically involves the reduction or removal of side chains or attached groups, or both, and the conjugation to other molecules such as glucose to form a glucuronide or conjugation with sulfate [40]. This finding seems to indicate an important correlation to the muscular dystrophy that affects this experimental group, especially because AfGR dogs usually cycle regularly and display the same clinical signs and behaviors as healthy dogs [41].

In summary, we have performed a comprehensive and quantitative monitoring of steroid profiles throughout the estrous cycle of normal and GRMD dogs. Significant differences in these profiles were observed between GRMD and healthy animals, most notably for estradiol. These findings contribute to a better understanding of both dog reproduction and the muscular dystrophy pathology. Hormonal behavior may affect the quality of life of DMD patients. An LC-MS/MS protocol was developed with isotope dilution and applied as a gold-standard primary method to accurately quantify the target steroids with very high sensitivity and appropriate figures of merit. The degree of precision achieved by this method for analyzing hormone levels allows for a statistical evaluation that correctly interrogates pathological status.

## References

[1] Valentine BA, Cooper BJ, Delahunta A, Oquinn R, Blue JT. Canine x-linked muscular-dystrophy—an animal-model of duchenne muscular-dystrophy—clinical-studies. J Neurol Sc. 1988;88: 69–81. 3225630 10.1016/0022-510x(88)90206-7

[2] Sharp NJH, Kornegay JN, Lane SB. The muscular-dystrophies. Sem Vet Med Surg-Sm Anim. 1989;4: 133–140. 2682886

[3] Hoffman EP, Brown RH, Kunkel LM. Dystrophin—the protein product of the duchenne muscular-dystrophy locus. Cell. 1987;51: 919–928. 3319190 10.1016/0092-8674(87)90579-4

[4] Cooper BJ, Winand NJ, Stedman H, Valentine BA, Hoffman EP, Kunkel LM, et al The homolog of the duchenne locus is defective in x-linked muscular-dystrophy of dogs. Nature. 1988;334: 154–156. 3290691 10.1038/334154a0

[5] Nguyen F, Cherel Y, Guigand L, Goubault-Leroux I, Wyers M. Muscle lesions associated with dystrophin deficiency in neonatal golden retriever puppies. J Comp Pathol. 2002;126: 100–108. 11944998 10.1053/jcpa.2001.0526

[6] Collins CA, Morgan JE. Duchenne's muscular dystrophy: animal models used to investigate pathogenesis and develop therapeutic strategies. Intern J Exp Pathol. 2003;84: 165–172. 14632630 10.1046/j.1365-2613.2003.00354.x PMC2517561

[7] Ambrósio CE, Fadel L, Gaiad TP, Martins DS, Araújo KP, Zucconi E, et al Identification of three distinguishable phenotypes in golden retriever muscular dystrophy. Genet Mol Res. 2009;8(2): 389–396. 19440974 10.4238/vol8-2gmr581

[8] Nghiem PP, Hoffman EP, Mittal P, Brown KJ, Schatzberg SJ, Ghimbovschi S, et al Sparing of the dystrophin-deficient cranial sartorius muscle is associated with classical and novel hypertrophy pathways in GRMD dogs. Am J Pathol. 2013;183(5): 1411–1424. 10.1016/j.ajpath.2013.07.013 24160322 PMC3814684

[9] Brinkmeyer-Langford C, Kornegay JN. Comparative Genomics of X-linked Muscular Dystrophies: The Golden Retriever Model. Curr Genomics. 2013;14(5): 330–342. 10.2174/13892029113149990004 24403852 PMC3763684

[10] Gaiad TP, Silva MB, Silva GC, Caromano FA, Miglino MA, Ambrósio CE. Physical therapy assessment tools to evaluate disease progression and phenotype variability in Golden Retriever muscular dystrophy. Res Vet Sci. 2011;91: 188–193. 10.1016/j.rvsc.2011.01.007 21315399

[11] Kerkis I, Ambrosio CE, Kerkis A, Martins DS, Zucconi E, Fonseca SAS, et al Early transplantation of human immature dental pulp stem cells from baby teeth to golden retriever muscular dystrophy (GRMD) dogs: Local or systemic? Journal of Translational Medicine. 2008;6:35 10.1186/1479-5876-6-35 18598348 PMC2529267

[12] Araujo KP, Bonuccelli G, Duarte CN, Gaiad TP, Moreira DF, Feder D, et al Bortezomib (PS-341) treatment decreases inflammation and partially rescues the expression of the dystrophin-glycoprotein complex in GRMD dogs. PLoS One. 2013;8(4):e61367 10.1371/journal.pone.0061367 23579193 PMC3620287

[13] Stanczyka FZ, Clarke, NJ. Advantages and challenges of mass spectrometry assays for steroid hormones. J Steroid Biochem Mol Biol. 2010;121: 491–495. 10.1016/j.jsbmb.2010.05.001 20470886

[14] Kushnir MM, Rockwood AL, Roberts WL, Yue B, Bergquist, J, Meikle AW. Liquid chromatography tandem mass spectrometry for analysis of steroids in clinical laboratories. Clin Biochem. 2011;44: 77–88. 10.1016/j.clinbiochem.2010.07.008 20627096

[15] Grebe SKG, Singh RJ. Clinical steroid mass spectrometry: LC-MS/MS in the clinical laboratory—where to from here? Clin Biochem Rev. 2011;32(1): 5–31. 21451775 PMC3052391

[16] Koala T, Schmiederera D, Pham-Tuana H, Röhringa C, Rauhb M. Standardized LC–MS/MS based steroid hormone profile-analysis. J Steroid Biochem Mol Biol. 2012;129: 129–138. 10.1016/j.jsbmb.2011.12.001 22210511

[17] Fortin T, Salvador A, Charrier JP, Lenz C, Bettsworth F, Lacoux X, et al Multiple reaction monitoring cubed for protein quantification at the low nanogram/milliliter level in nondepleted human serum. Anal Chem. 2009;81: 9343–9352. 10.1021/ac901447h 19839594

[18] Janssen PM, Murray JD, Schill KE, Rastogi N, Schultz EJ, Tran T, et al Prednisolone attenuates improvement of cardiac and skeletal contractile function and histopathology by lisinopril and spironolactone in the mdx mouse model of Duchenne muscular dystrophy. PLoS One. 20114;9(2): e88360 10.1371/journal.pone.0088360 24551095 PMC3923790

[19] Brolio MP, Cima DS, Miglino MA, Ambrosio CE. Histological comparison of the smooth uterine muscle of healthy golden retriever bitches, carriers of the progressive muscular dystrophy (GRMD) gene, and GRMD-affected bitches. Anim Reprod Sci. 2014;150(10): 56–61. 25200710 10.1016/j.anireprosci.2014.08.005

[20] EC 2002. Commission Decision 2002/657/EC of 12 August 2002 implementing Council Directive 96/23/EC. Off J Europ Commun. L 221:8–36.

[21] Liang HR, Foltz RL, Meng M, Bennett P. Ionization enhancement in atmospheric pressure chemical ionization and suppression in electrospray ionization between target drugs and stable isotope-labeled internal standards in quantitative liquid chromatography/tandem mass spectrometry. Rapid Commun. Mass Spectrom. 2003;17: 2815–2821. 14673832 10.1002/rcm.1268

[22] Giton F, Caron P, Bérubé R, Bélanger A, Barbier O, Fiet J, et al Plasma estrone sulfate assay in men: comparison of radioimmunoassay, mass spectrometry coupled to gas chromatography (GC-MS), and liquid chromatography-tandem mass spectrometry (LC-MS/MS). Clin Chim Acta. 2010;411: 1208–1213. 10.1016/j.cca.2010.04.022 20427015

[23] Etter ML, Eichhorst J, Lehotay DC. Clinical determination of 17-hydroxyprogesterone in serum by LC-MS/MS: Comparison to Coat-A-CountTM RIA method. J Chromatog B. 2006;840: 69–74. 16737854 10.1016/j.jchromb.2006.04.038

[24] Gust M, Vulliet E, Giroud B, Garnier F, Couturier S, Garric C, et al Development, validation and comparison of LC-MS/MS and RIA methods for quantification of vertebrates-like sex-steroids in prosobranch molluscs. J Chromatog B. 2010;878: 1487–1492. 10.1016/j.jchromb.2010.03.046 20399713

[25] Koal T, Schmiederer D, Pham-Tuan H, Röhring C, Rauh M. Standardized LC-MS/MS based steroid hormone profile-analysis. J Steroid Biochem Mol Biol. 2012;129: 129–138. 10.1016/j.jsbmb.2011.12.001 22210511

[26] Fanelli F, Belluomo I, Di Lallo VD, Cuomo G, De Iasio R, Baccini M, et al Serum steroid profiling by isotopic dilution-liquid chromatography-mass spectrometry: Comparison with current immunoassays and reference intervals in healthy adults. Steroids. 2011;76: 244–253. 10.1016/j.steroids.2010.11.005 21112348

[27] Hydbring-Sandberg E, von Walter LW, Hoglund K, Svartberg K, Swenson L, Forkman B. Physiological reactions to fear provocation in dogs. J. Endocrinol. 2004;180: 439–448. 15012598 10.1677/joe.0.1800439

[28] Jones AC, Josephs RA. Interspecies hormonal interactions between man and the domestic dog (Canis familiaris). Horm Behav. 2006;50: 393–400. 16784746 10.1016/j.yhbeh.2006.04.007

[29] Guyton AG, Hall JE. (2006) Tratado de Fisiologia Médica Hormônios Adrenocorticais Elsevier, Rio de Janeiro, Chapter11:944–960.

[30] Nozoe KT, Hachul H, Hirotsu C, Polesel DN, Moreira GA, Tufik S, et al The relationship between sexual function and quality of sleep in caregiving mothers of sons with duchenne muscular dystrophy. Sex Med. 2014;2(3): 133–140. 10.1002/sm2.29 25356310 PMC4184493

[31] Beerda B, Schilder MBH, Bernadina W, Van Hooff J, De Vries HW, Mol JA. Chronic stress in dogs subjected to social and spatial restriction. II. Hormonal and immunological responses. Physiol Behav. 1999;66: 243–254. 10336150 10.1016/s0031-9384(98)00290-x

[32] Goy-Thollot I, Decosne-Junot C, Bonnet JM. Influence of aging on adrenal responsiveness in a population of eleven healthy beagles. Res Vet Sci. 2007;82: 195–201. 17011003 10.1016/j.rvsc.2006.07.010

[33] Ghaffari MS, Dezfoulian O, Aldavood SJ, Masoudifard M. Estrogen-related alopecia due to polycystic ovaries in a terrier dog. Comp Clin Pathol. 2009;18: 341–343.

[34] Pfaff DW, Phillips MI, Rubin RT. (2004) Hormone metabolites can be the behaviorally active compounds In: Principles of hormonal/behavioral relations. Rijnberk A., Kooistra HS: Elsevier Academic Press, California, United States, 61–65.

[35] Inaba T, Namura T, Tani H, Matsuyama S, Torii R, Kawate N. Enhancement of aromatase gene expression in the mediobasal hypothalamus during estrus in the beagle bitch. Neurosci Lett. 2002;333: 107–110. 12419492 10.1016/s0304-3940(02)01001-7

[36] Morini AC, Brolio MP, Millano A, Braggio LZ, Martins DS, Perecin F., et al Existem diferenças nos parâmetros hematológicos e bioquímicos séricos entre fêmeas normais e portadoras do modelo experimental GRMD (Golden Retriever Muscular Dystrophy)? Pesq Vet Bras. 2011;31: 94–98.

[37] Stapleton DI, Lau X, Flores M, Trieu J, Gehrig SM, Chee A. Dysfunctional muscle and liver glycogen metabolism in mdx dystrophic mice. PLoS One. 2014;9(3):e91514 10.1371/journal.pone.0091514 24626262 PMC3953428

[38] Feldman EC, Nelson RW. (2003) Canine and feline endocrinology and reproduction, W.B.Saunders, Philadelphia, 1089 p.

[39] Bush BM (2004). Interpretação dos resultados laboratoriais para clínicos de pequenos animais Rocca, São Paulo, 376 p.

[40] David ON (2007) Vertebrate Endocrinology Elsevier Academic Press, USA, 531 p.

[41] Peres MA, da Rocha AM, Vannucchi CI, Mendes CM, Cavalcanti PV, Nichi M., et al Semen analysis of Golden Retriever healthy dogs and those affected by muscular dystrophy. Andrologia. 2014;46(3): 277–282. 10.1111/and.12079 23463904

